# Supplementary figures and images for: The pathway of unconventional protein secretion involves CUPS and a modified trans-Golgi network
Source: J Cell Biol. 2025 Feb 27;224(5):e202312120. doi: 10.1083/jcb.202312120 (PMC11867701; doi:10.1083/jcb.202312120)

Source Data Fig 3

A

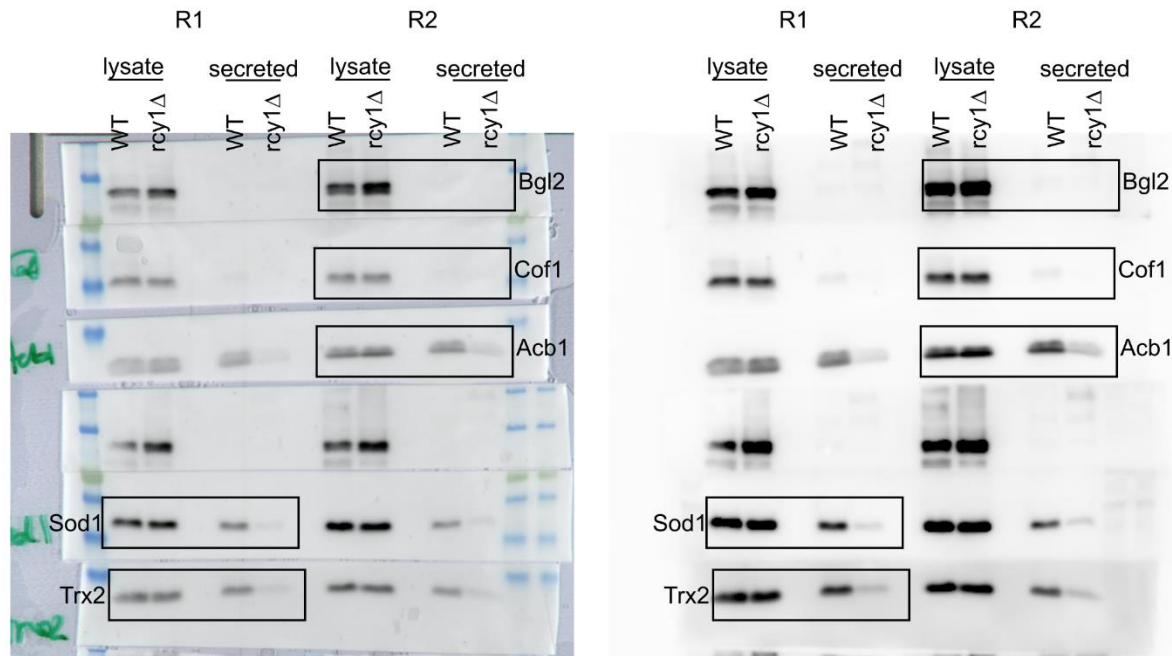

B

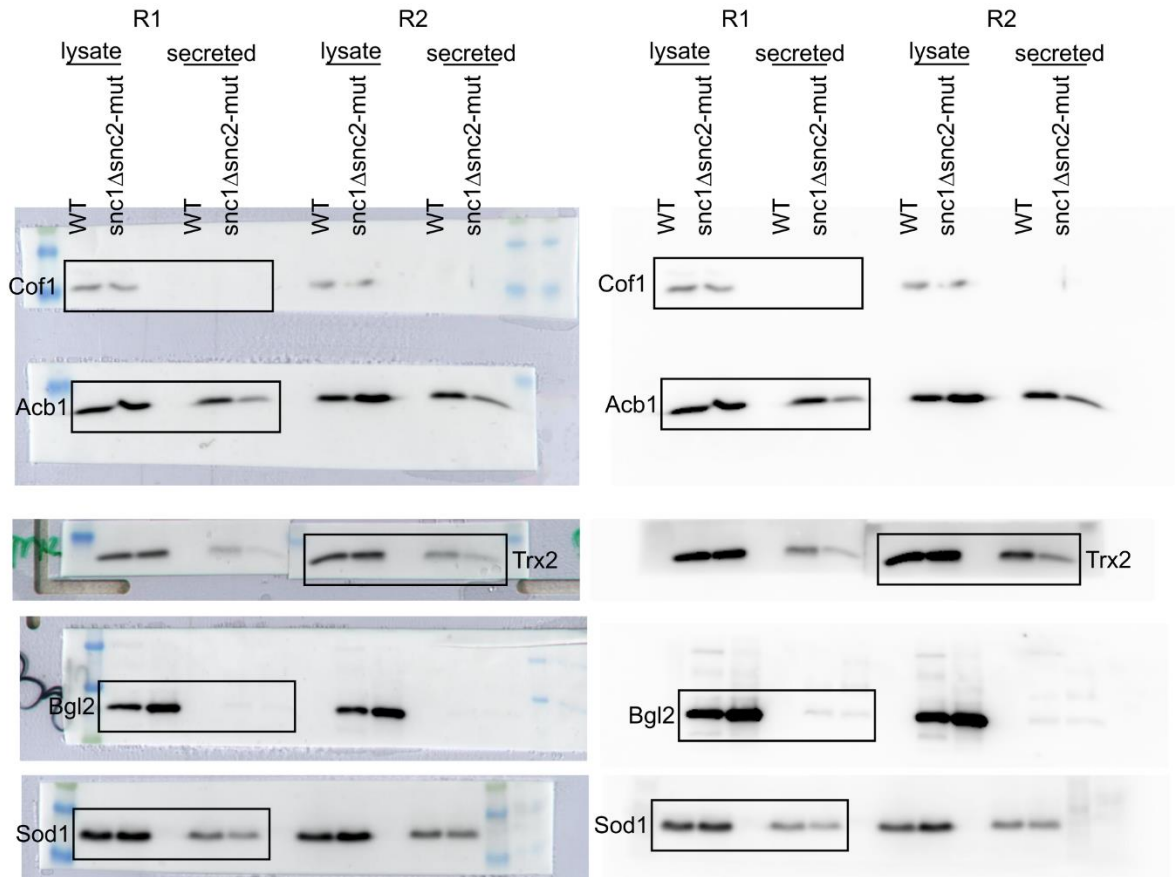

Supplement: SourceData F3 — is the source file for Fig. 3. [file jcb_202312120_sourcedataf3.pdf]
